# Daily Stress Processes in a Pandemic: The Effects of Affect, Worry, and Age

**DOI:** 10.1093/geroni/igaa057.3446

**Published:** 2020-12-16

**Authors:** Niccole Nelson, Cindy Bergeman

**Affiliations:** University of Notre Dame, Notre Dame, Indiana, United States

## Abstract

On March 13th, 2020, the World Health Organization declared a novel coronavirus, COVID-19, a pandemic. Given the day-to-day behavioral changes necessitated by this global threat, the current study examined daily stress reactivity and its potential moderators during the COVID-19 pandemic. Two-level, multilevel modeling was used to examine the daily relationship between perceived stress and negative affect, as well as the moderating effects of daily positive affect, average pandemic worry, and age, on this process. Participants included 349 individuals from the young adult, midlife, and later-life cohorts of the Notre Dame Study of Health & Well-being who completed a 28-day, daily diary study amidst the COVID-19 pandemic (NDHWB; Age Range = 26-89). Individuals were affectively reactive to perceived stress during the COVID-19 pandemic, experiencing higher negative affect on days of higher perceived stress. Regarding moderators, older individuals were less stress reactive than younger individuals, and the extent of individuals’ pandemic worry exacerbated their stress reactivity. Furthermore, daily positive affect buffered daily stress reactivity, regardless of pandemic worry and age. In sum, individuals who were younger or more worried about the pandemic tended to be more stress reactive than older or less worried individuals. Furthermore, daily positive affect buffered stress reactivity, and this buffering effect did not depend on age or the extent to which individuals were worried about the pandemic. Thus, mobilizing positive affect during the COVID-19 pandemic may be a promising avenue for intervention in daily stress processes.

